# Green Lacewing *Chrysoperla externa* Is Attracted to Volatile Organic Compounds and Essential Oils Extracted from *Eucalyptus urograndis* Leaves

**DOI:** 10.3390/plants13162192

**Published:** 2024-08-08

**Authors:** David Jackson Vieira Borges, Rafael Aparecido Carvalho Souza, Alberto de Oliveira, Raquel Maria Ferreira de Sousa, Henrique Venâncio, Guilherme Ramos Demetrio, Bianca Giuliano Ambrogi, Jean Carlos Santos

**Affiliations:** 1 Pos-Graduate Program in Ecology, Conservation and Biodiversity, Federal University of Uberlandia, Uberlandia 38405-240, Minas Gerais, Brazil; borges.djv@gmail.com; 2 Institute of Chemistry, Federal University of Uberlândia, Uberlândia 38408-100, Minas Gerais, Brazil; rafasouza27@hotmail.com (R.A.C.S.); alberto@ufu.br (A.d.O.); rsousa@ufu.br (R.M.F.d.S.); 3 Pos-Graduate Program in Ecology and Conservation, Federal University of Sergipe, São Cristóvão 49107-230, Sergipe, Brazil; henrivens@gmail.com; 4 Laboratory of Plant Ecology, U. E. Penedo, Campus Arapiraca, Federal University of Alagoas, Penedo 57200-000, Alagoas, Brazil; guilherme.ferreira@penedo.ufal.br; 5 Department of Ecology, Federal University of Sergipe, São Cristóvão 49107-230, Sergipe, Brazil; biancaambrogi@academico.ufs.br

**Keywords:** chemical composition, eucalyptol, α-terpinyl acetate, olfactometer, plant predator interactions, simulated herbivory

## Abstract

Plant herbivore interactions have long been recognized as a complex interplay influenced by various factors, including plant volatile emissions. Understanding the role of these volatiles in mediating plant predator interactions is crucial for developing sustainable pest management strategies. This study investigated the olfactory preferences of *Chrysoperla externa* larvae for volatiles emitted by *Eucalyptus urograndis* leaves, focusing on both seedlings and essential oils (EOs). We used Y-tube olfactometry to compare larval preferences between the clean air and various plant treatments, including undamaged and herbivore-damaged leaves. Chemical analysis of EOs revealed higher concentrations of oxygenated monoterpenes and sesquiterpenes in young and damaged leaves, particularly linalool, which has been implicated in insect attraction. Our results showed a significant preference for volatiles emitted by young damaged leaves over clean air for both seedlings (χ^2^ = 11.03, *p* = 0.001) and EOs (χ^2^ = 9.76, *p* = 0.002). *Chrysoperla externa* larvae are significantly attracted to specific volatiles from damaged *E. urograndis* leaves, suggesting these compounds could serve as cues for natural enemy foraging. Our findings enhance the understanding of plant–predator dynamics and suggest potential applications of eucalyptus plantations to sustain *C. externa* populations for biocontrol purposes.

## 1. Introduction

Plants are sessile organisms that are evolutionarily selected to invest their limited resources in a functionally equilibrated way [1], in the sense that the most limiting resources will drive biomass and carbon allocation. However, a considerable amount of this carbon does not remain fixed in the plant and is emitted back into the atmosphere in the form of volatile organic compounds (VOCs) [2]. Studies on VOCs have received more attention in recent years [3] and have indicated that these compounds play major roles in plant communication and signaling abilities, participating in complex processes such as plant plant [4], plant microbe [5], and plant insect interactions [6]. In the latter case, owing to their tight coordination needs, pollination and herbivory can be highlighted as strongly dependent on these signals [2,7].

The production and emission of these compounds are strongly related to plant fitness, as they can alter the dynamics of plant interactions, enhance flower appearance to pollinators [8], or indicate to natural enemies where to find herbivore prey [9]. In this sense, VOCs can be released during the interaction between a plant and its herbivore (Herbivore-Induced Plant Volatiles—HIPV) and are pointed as indirect defenses for plants based on their function of herbivores natural enemies’ attraction [10]. HIPVs are identified as a multiple communication strategy and may represent signaling pathways among plants, plant parts, and plant and natural enemies [11]. In these cases, when inducible defenses are expressed in herbivore presence conditions, the cost of production of these compounds would be surpassed by their benefits, as they would imply a herbivory decrease [12].

The emission and composition of HIPVs depend on many factors and may be influenced by the intrinsic and ecological conditions of the plant. For example, genotypes within a species may present different VOC emission profiles [13], and their profiles and compositions are strongly genotype-dependent [14]. Another important driver of VOC production is the organ or individual ontogenetic stage [15]. The optimal defense theory predicts that induced defenses are more prominent in organs with higher fitness values [16], such as young tissues [17]. Abiotic conditions may also be a strong driver of plant VOC profiles, as convergent evolution appears in some volatile production along plant phylogeny [18]. Biological interactions can also trigger different HIPVs emissions, because the emission of these compounds is elicited by specific herbivory types [19].

Plant-derived odors can be obtained using essential oils (EOs), which contain a variety of compounds (e.g., alkaloids, flavones, lignans, and phenols), some of which are volatile organic compounds (VOCs) [20,21,22]. These essential oils are highly volatile secondary metabolites that are distilled from plants and may play a significant role in plant–insect interactions, such as repelling insects (e.g., [23]). There is evidence for animals avoiding EOs in preference studies [24] and showing preferences for feeding on plants with lower EO concentrations [25]; these compounds have been shown to be a good proxy for the VOC emission profile of a plant [26]. For example, many important monoterpenes and sesquiterpenes emitted as HIPVs are stored in EOs [27]. The emission mechanism of VOCs is complex and may depend on their volatility and cytological excretory processes [20]. Understanding the relationship between EO composition, VOCs, and natural enemies is a cornerstone for understanding the interactions among plants, herbivores, and their natural enemies [28]. Knowledge regarding the ecological functions and mechanisms of herbivore-induced plant volatile (HIPV) emissions would allow the development of new integrated strategies to biological control programs to enhance the ability of natural enemies to effectively suppress pest populations in productive systems [29]. Among the plant species with promising essential oils used as repellents, *Eucalyptus* spp. are the most cited in specialized literature [30].

In Brazil, the *Eucalyptus* production system is a good model for testing the importance and relevance of EOs as insect repellents or attractants. Data from the Brazilian Tree Industry (IBÁ) show that the reforestation area corresponds to 9.9 million hectares of planted trees with *eucalyptus*, pine, and other forest species (acacia, rubber, paricá, and teak) [31]. These trees are used for cellulose and paper, steel and charcoal, wood panels, laminate flooring, and other purposes [31,32]. In 2022, this sector was responsible for BRL 244.6 billion in gross revenues, for BRL 25.3 billion in federal, and for 2.6 million jobs, both direct and indirect [31]. Several insects, such as leaf-cutting ants (*Atta* spp. and *Acromyrmex* spp.), termites of the genera *Coptotermes* spp. and *Heterotermes* spp., and the defoliating caterpillars (*Eacles imperialis magnifica)* (Walker, 1856) (Lepidoptera: Saturniidae); as well as introduced species, such as the bronze bug, *Thaumastocoris peregrinus* (Carpintero & Dellapé, 2006) (Hemiptera: Thaumastocoridae), the gall wasps, *Epichrysocharis burwelii* (Schauff) (Hymenoptera: Eulophidae) and *Leptocybe invasa* (Fisher and La Salle, 2004) (Hymenoptera: Eulophidae), and the psyllid, *Ctenarytaina spatulate* (Taylor, 1997) (Hemiptera) [33] have adapted to *Eucalyptus* plantations. This is probably due to the similarity of these plants with other native Brazilian species and the homogeneity of planting, which is a constant source of food [33,34]. Chrysopidae species are among the natural predators used in the biological control of insects and pests in *Eucalyptus* forests [35]. Within the green lacewing species, *Chrysoperla externa* (Hagen, 1861) is a predator with a wide geographical distribution that thrives in a great variety of habitats and is considered one of the most promising species for biological pest control [36,37]. Thus, to understand the interactions between predatory natural enemies and forest tree species, this study aimed to verify whether the volatiles emitted by young, mature, damaged, and undamaged leaves of *E. urograndis* act as attractants for chrysopid larvae.

We analyzed the chemical composition of the EO from *E. urograndis* leaves and hypothesized that there would be a difference in the essential oil composition between damaged and undamaged young and mature leaves of *E. urograndis*. We hypothesized that chrysopid larvae prefer the volatiles of young leaves subjected to injury using seedlings and EOs, considering that they present a higher herbivory probability [38] and a higher production of VOCs as sesquiterpenes [39], which attract predators. We expect that the observations obtained from the experimental analyses will provide a basis to list the compounds that attract or repel *C. externa* individuals, resulting in a deeper understanding of the behavior of this natural predator used in the biological control of eucalyptus forests.

## 2. Materials and Methods

### 2.1. Study System

*Eucalyptus* is a genus that originates in Australia, Tasmania, and other islands in Oceania, with approximately 730 species, of which only 20 are currently used for commercial purposes worldwide [40]. *Eucalyptus urograndis*, a hybrid of *E. grandis* and *E. urophylla*, is one of the most used clone species in Brazil [41]. This combination resulted in vigorous trees with wood of greater density and great resistance to cancer caused by the fungus *Cryphonectria cubensis* (Bruner) Hodges, 1980 (Diaporthales: Cryphonectriaceae) [41,42]. 

Chrysopidae species are among the natural predators used in the biological control of insects and pests in *Eucalyptus* forests and are the second-largest family of Neuroptera, with 75 genera, 11 subgenera, and 1200 species [43]. Chrysopids, also called green lacewings, are known as trash-carriers because the larvae of many species carry debris on their backs, which gives them protection against natural enemies through physical barriers and camouflage. Debris consists of exoskeletons from their prey, fibers of plant or animal origin, and other particles encountered during their movement. They are attached to the body by numerous long, smooth, or serrated bristles with a straight or hook-shaped tip that exists on the dorsal surface and lateral tubercles of the thorax and abdomen [44]. The larvae of *C. externa* have great reproductive and locomotion capacity and tolerance to insecticides, and they can feed on a wide variety of arthropods, including mites, and small phytophagous insects such as aphids [45]. 

Knowledge of the life cycle and its assertiveness in the search for prey and adaptation to different climatic conditions, combined with the development of diets and mass creation in the laboratory, is reflected in the increased research interest in *C. externa* and the Neuroptera group in general for application in biological control programs [46,47,48,49,50].

### 2.2. Plant Culture

*Eucalyptus urograndis* clones (Urograndis I144 type) were purchased from a plant nursery, “Viveiro Valor Verde” (Araguari, Minas Gerais, Brazil; 18°39′29.3″ S and 48°09′09.4″ W). All plants were cultivated under similar conditions [substrate (50% local soil and 50% cattle manure), irrigation (once a day), and temperature (environmental temperature)]. The experiments were carried out with *E. urograndis* seedlings that were 70 days old, ca. 30 cm high, and contained 10–12 expanded leaves. These plants did not present any herbivory damage or necrosis on their leaves before the experiments.

### 2.3. Simulated Herbivory Treatment

The simulation of herbivory on *E. urograndis* leaves was performed using an artificial damage technique by removing 6 mm leaf disc diameters using a hole punch (model P202, Tilibra, Bauru, Brazil), selected plants with leaves without any sign of apparent and/or significant damage (<1% of leaf area) from herbivory and/or pathogens. This represented a reduction ranging from 10 to 15% in the area of each leaf. Holes were not made in the midribs of the leaves. The plants were divided into three groups: (1) seedlings without damaged leaves; (2) seedlings with young damaged leaves, comprising plants in which we made four to five holes in four leaves located at the top of the seedling; and (3) seedlings with mature leaves with damage, composed by plants in which we made four to five holes in four leaves located at the bottom of the seedling.

Based on the methodology of Resende et al. [51], a previous study on the displacement of air inside a Y-tube was conducted using water and dry ice. Our results showed that there was no mixing of the air between the arms. For each bioassay, one *C. externa* larva was inserted at the base of the olfactometer. The larvae were used only once, and each time, another larva was selected for a new test. The choice was made when the larva entered more than 1/3 of the Y-tube arm and remained there for 15 s. The maximum time required for bioassay was 10 min. Only the larvae that fulfilled these criteria were considered in the statistical analyses. We performed a total of 409 and 328 bioassays for seedlings and EO, respectively; 280 (68.46%) and 229 (69.82%) larvae behaved according to the established criteria, and 129 and 99 were not considered for the final dataset as they did not respond to the established criteria for seedlings and EO, respectively. Therefore, for statistical analysis, we used 40 larvae in each of the seven combinations (n = 280 larvae) for seedlings, and between 30 and 37 larvae in each of the seven combinations (n = 229 larvae) for EO.

To avoid any positional effects, the Y-tube was horizontally turned 180° (clockwise direction) after each tested larva, and the odors on the presenting side were changed every three assays. The Y-tube was then changed after six assays. Plants were changed every 12 assays, as the production of volatiles can vary among individuals [52]. In all analyses, although the sources of odor and the arms of the olfactometer were inverted to avoid any bias, an additional test was performed with the provision of clean air in both arms. The results of this test demonstrated that there were no defects on either side of the olfactometer, proving the efficiency of the system and corroborating the results of other studies that have used similar methodologies. Tests of this nature are commonly performed in experiments with an olfactometer system to ensure that no bias occurs, for example, Blassioli-Moraes et al. [53] used the same methodology to evaluate the response of the parasitoid *Telenomus podisi* (Ashmead) (Hymenoptera: Scelionidae) to the volatiles of soybean seeds *Glycine max* (L.) Merrill and nymphs of *Euschistus heros* (F.) (Heteroptera: Pentatomidae). After every 12 assays, the Y-tube and glass chambers were washed with neutral detergent, water, and 70% ethanol and placed in an oven at 100 °C for 60 min. The assays were performed from 7 am to 5 pm under constant laboratory conditions of luminosity (fluorescent light) and temperature (~25 °C). 

The behavioral responses of *C. external* larvae to *E. urograndis* volatiles in the olfactory system were evaluated using seedlings and EO for seven combinations as follows: (i) clean air in both arms of the olfactometer; (ii) undamaged plants versus clean air; (iii) young leaves with damage versus clean air; (iv) mature leaves with damage versus clean air; (v) young leaves with damage versus undamaged plants; (vi) mature leaves with damage versus undamaged plants; and (vii) young leaves with damage versus mature leaves with damage. 

### 2.4. EO Extraction

We extracted essential oil (EO) of *E. urograndis* leaves from each of the four groups of leaves (young with and without damage, mature with and without damage). Simulated herbivory damage was performed on leaves of damaged groups 60 min before the oil extraction [54]. We initially measured the moisture content of fresh leaves to accurately calculate the EO yield. We used the gravimetric method for this procedure utilizing an infrared moisture determination balance (Kett FD-600, Kett Electric Laboratory Co., Ltd., Tokyo, Japan). This was done with 1.0 g of leaves, which were exposed to a temperature of 105 ± 5 °C until the achievement of constant biomass. In sequence, a Clevenger apparatus was used to extract EO from the leaves, through hydrodistillation, under reflux for 4 h with 50 g of fresh leaves [55]. The extraction was performed in triplicate. The EO was extracted from water with dichloromethane (Vetec, Rio de Janeiro, RJ, Brazil) (3 × 10 mL), and the solvent was removed via evaporation at 35 °C. Finally, leaf EOs were stored in glass bottles, sealed, and refrigerated in the dark.

### 2.5. EO Chemical Composition

We used a gas chromatograph coupled to a mass spectrometer (GC-MS) (Shimadzu, QP2010 model, Kyoto, Japan) using a DB-5 capillary column (J&W, 30 m × 0.25 mm × 0.25 m, Tuxedo Park, NY, USA) to identify the composition of the extracted EOs. EO samples were solubilized in dichloromethane (5 mg mL^−1^). We applied the following conditions: helium as a carrier gas with a constant flow of 1.02 mL min^−1^, injector temperature of 240 °C, detector temperature of 220 °C, splitless mode of injection (1:10), oven temperature programmed to initiate at 60 °C and increase to 246 °C at a rate of 3 °C min^−1^, an ionizing potential of 70 eV, and a range of *m/z* 40–650 [56]. Compound identification of the EO was based on the similarity index (SI) obtained using the software (LabSolution version GC-MS) with the mass spectral commercial libraries Nist27, Nist147, Wiley7, Wiley229, and Shim2205. The arithmetic index (AI) was also used to identify compounds in standard alkanes [53]. AI was calculated using the equation AI (x) = 100 C (Pz) + 100 [(RT (x) − RT (Pz))/(RT (Pz + 1) − RT (Pz))], where RT is the retention time in min, x is an unknown compound, C is the carbon number of the alkane Pz that runs before x, and Pz + 1 is the alkane that runs after x. The AI obtained was compared with the AIs of the Webbook-NIST Standard Reference Data [56] and Adams Book [57].

### 2.6. Insect Culture

*Chrysoperla externa* larvae were obtained from the ALB Agroambiental biofactory (Uberlândia, Minas Gerais, Brazil). Rearing was performed according to the methodology described by Macedo and Soares [58]. The larvae were obtained from *C. externa* adults, which were collected in the field, sent to the biofactory, and placed in cages. The laboratory environment was maintained under a controlled temperature (25 ± 2 °C), air humidity (70 ± 10%), and photoperiod of 14:10 h (light:dark). Plastic cages were prepared from polyvinyl chloride (PVC) pipes (segments of 23 cm × 23 cm) sealed at the top and bottom with an organza fabric. The cage was internally lined with a sheet of A4 paper to allow the removal of eggs. We inserted approximately 12 adult couples of *C. externa* into each cage and fed them with cotton containing an aqueous solution of yeast and honey through the upper part of the cage. Some of the collected eggs were reserved to restart the *C. externa* rearing cycle. The remaining eggs were transferred to a plastic container containing *Anagasta kuehniella* eggs (Zeller, 1879) (Lepidoptera: Pyralidae) to feed the hatched *C. externa* larvae. The experiments described below were performed with *C. externa* larvae of 7 ± 2 days of age.

### 2.7. Behavioral Evaluation Using Y-Tube Olfactometer System

**Figure 1:**
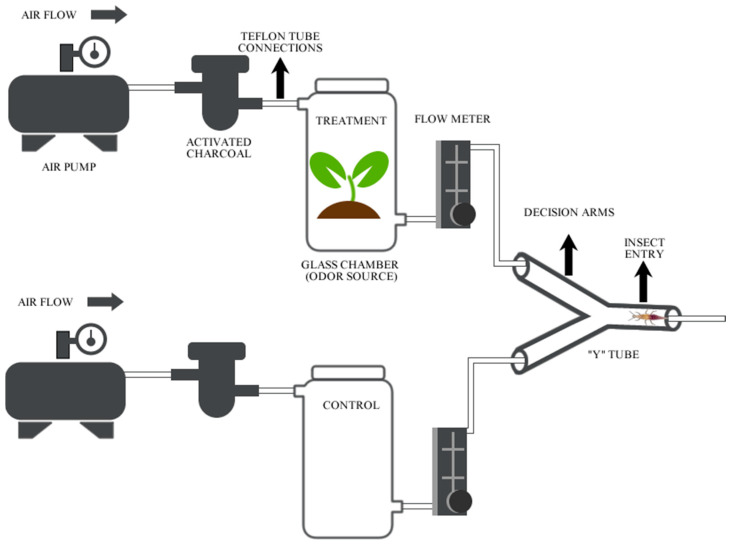
Schematic representation of the Y-tube olfactometer system used in the experiment to evaluate the olfactory response of the larvae of *Chrysoperla externa* to volatile compounds or essential oils (EO) emitted by the leaves of *Eucalyptus urograndis*.

The olfactory response of *C. externa* to volatile organic compounds emitted by the leaves and EO of *E. urograndis* was evaluated using a Y-olfatometer model modified from Akol and Njagi [59], Du et al. [60], and Han and Chen [61] (Figure 1). For the analysis using seedlings, the tubes with the plants were wrapped with aluminum foil and closed to the height of the stem to avoid contamination of the air with volatile compounds from the plastic of the tube, from the substrate used to grow the plants, or from the microorganisms present in the substrate [62,63]. For the analysis using EO, 0.1 g was deposited in 5 mL glass vials. The plants and EO were placed in the glass clamber of the system. The Y-tube olfactometer was composed of two arms at an angle of 120°. Each arm had a length of 17 cm and an internal diameter of 2 cm. The airflow inside the Y-tube was generated by using a vacuum pump (model A320, Big Air, Laguna Hills, CA, USA). The air initially flows through an activated carbon filter (Fit 200 model, Planeta Água, São Paulo, Brazil) to purify and remove impurities, and is then forced through a glass chamber (8.5 cm × 23 cm), where the plants were placed. The entire system was interconnected by PTFE tubes. An airflow of 1.5 L min^−1^ was used in the system, and the flow was controlled using two flow meters (LPM Air model, Key Instrumental, Croydon, PA, USA). We used an airflow of 1.5 L min^−1^ based on a previous behavioral study with *Chrysoperla externa* (see Resende et al. [51]).

### 2.8. Data Analysis

We applied chi-square (χ^2^) tests to evaluate the distribution of the percentage of choice between the alternatives of each olfactometry test, considering the expected proportion of choice as 0.50 (50%) for each arm of the Y-tube olfactometer as the null hypothesis. The percentage of choice was calculated as the ratio between the number of times that *C. externa* larvae chose a determined condition offered in one of the Y-olfactometer arms and the total number of assays performed for each combination. We used Spearman’s Correlations to verify the relationship between the proportions of choice between seedlings and EOs for the right and left sides, which allowed us to verify the linkage between VOCs emitted by the seedlings and the stored EOs. All analyses were performed in R [64], with a significance level of 95% (α = 0.05).

## 3. Results

### 3.1. Essential Oil Extraction

The extraction yield and amount of EO from *E. urograndis* leaves obtained by hydrodistillation are shown in Table 1. The yield obtained in this study ranged from 0.29% mature leaf biomass to 0.50% young leaf biomass.

### 3.2. Essential Oil Chemical Composition

**Figure 2:**
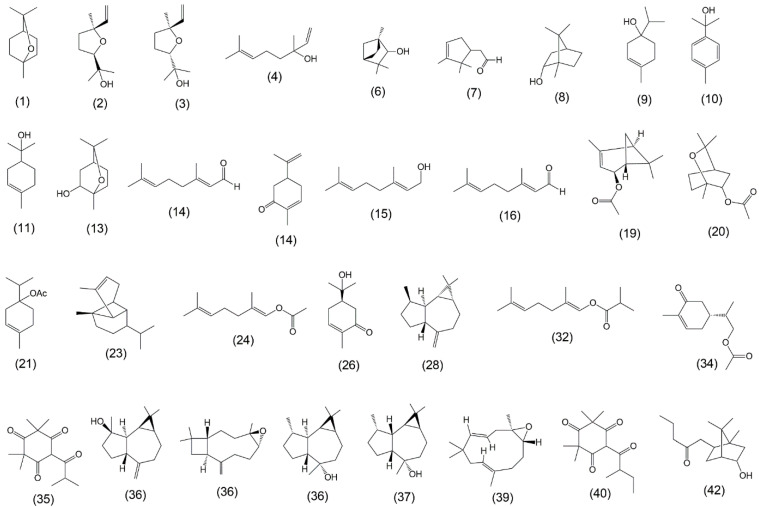
Structures of compounds identified in *Eucalyptus urograndis* essential oils.

The chemical composition of EOs from each group of *E. urograndis* leaves was determined using GC-MS and chromatogram profiles (Figure S1) showed 42 peaks, enabling the identification of 32 compounds. The AI values of the identified EO are listed in Table S1. Table 2 shows the percentage of compounds identified by the total ion chromatogram. Figure 2 shows the structures of the identified compounds, of which 69% are oxygenated monoterpenes and 22% are oxygenated sesquiterpenes. Eucalyptol, linalool, borneol, α-terpineol, neral, carvone, geraniol, and α-terpinyl acetate are the major oxygenated monoterpenes. Alpha-copaene, aromadendrene, spathulenol, caryophyllene oxide, globulol, and viridiflorol were the major sesquiterpenes.

According to Table 2, compounds eucalyptol, α-terpineol, α-terpinyl acetate, and caryophyllene oxide had the highest concentrations of all EOs. There was a higher concentration of spathulenol (12.20%), α-terpineol (11.9%), and eucalyptol (10.36%) in the EO of young leaves damaged. The essential oils of the damaged mature leaves contained 12.37% spathulenol, which was higher than that of eucalyptol (7.59%). However, the chromatogram analysis indicated that this peak was a mixture of spathulenol, caryophyllene oxide, and globulol, which are all oxygenated sesquiterpenes. The sum of these compounds is representative, accounting for almost 50% of the EO mass.

Table 3 shows the percentage variation in the main compounds of the EOs of young and mature leaves damaged by *E. urograndis*. The compounds aromadendrene and 5-hydroxy-isobornyl-isobutanoate, although having a low concentration in the total ion chromatogram (TIC), are among the 16 main compounds identified, as they make up about 80% of the composition of the EOs of *E. urograndis*. These two compounds caused a significant change in the relative composition of the EO from the damaged leaves, and the EOs of damaged young and mature leaves showed an increase in the concentration of these compounds in undamaged leaves. The essential oils of damaged leaves increased the production of both α-terpineol (9.6 and 19.5% for young and mature leaves, respectively) and α-terpinyl acetate (47.9 and 25% for young and mature leaves, respectively).

In contrast, damaged leaves showed a significant decrease in eucalyptol production (−63.2 and −62.7% in young and mature leaves, respectively). The essential oils of damaged young leaves resulted in an increase of 50.0% in aromadendrene and 39.7% in 5-hydroxy-isobornyl-isobutanoate content. The essential oils of mature damaged leaves resulted in an increase of 44.9% in aromadendrene and a reduction of 24.8% in 5-hydroxy-isobornyl-isobutanoate.

### 3.3. Olfactometry Tests

**Figure 3:**
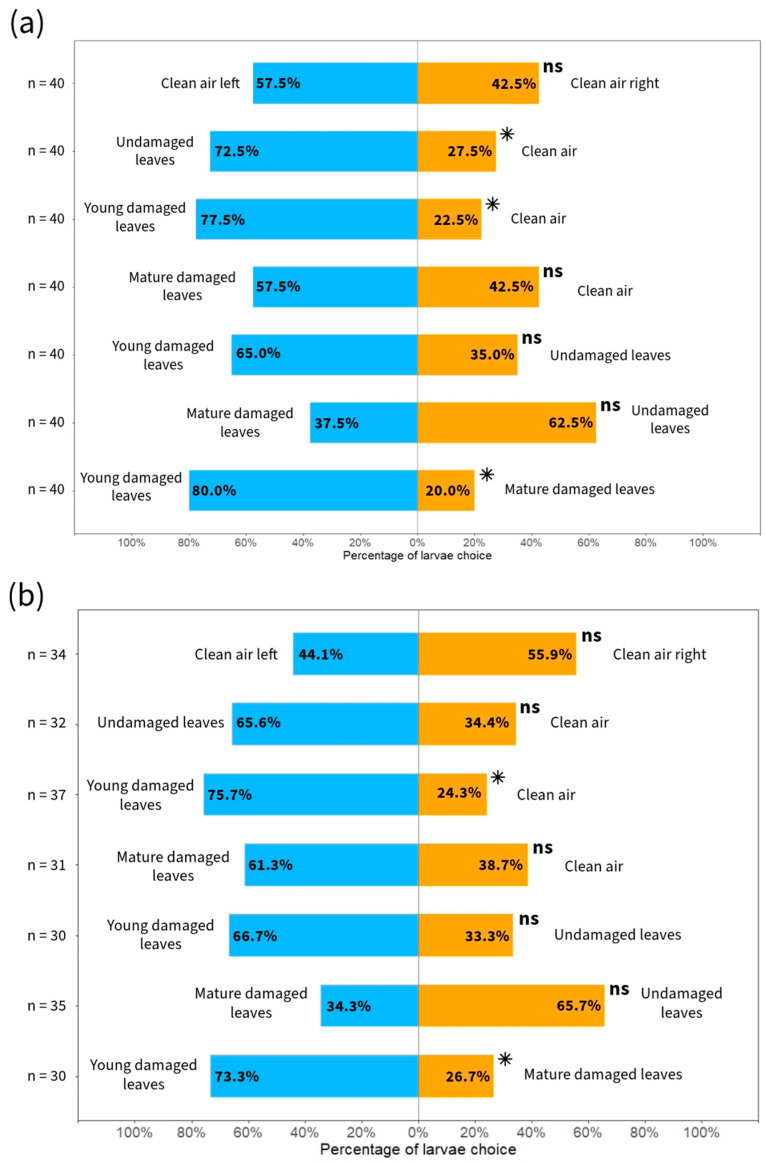
Frequency distribution of *Chrysoperla externa* larval choice to *Eucalyptus urograndis* leaves at different ontogenetic stages and simulated herbivory using seedlings (**a**) and essential oils extracted from these groups (**b**). n = number of larvae that chose one of the arms; ns: not significant; * *p* < 0.05.

In the treatment in which clean air was offered on both sides of the Y-tube olfactometer, the larvae randomly selected both the left and right sides, and there was no significant difference (for seedlings, $\chi_{1}^{2}=0.63$, p=0.429; and for EO, $\chi_{1}^{2}=0.47$, p=0.493; Figure 3). When compared to clean air, the larvae preferred the volatile compounds of undamaged leaves for seedlings ($\chi_{1}^{2}=7.23$, p=0.007), but not for EO ($\chi_{1}^{2}=3.125$, p=0.077), and individuals with young damaged leaves (for seedlings $\chi_{1}^{2}=11.03$, p=0.001; and for EO $\chi_{1}^{2}=9.76$, p=0.002; Figure 3). The frequency of larval choice for one side of the Y-tube was greater than 70% in these tests. However, in addition to the higher frequency of choice for seedlings with mature damaged leaves regarding clean air (for seedlings 57.5 versus 42.5%, $\chi_{1}^{2}=0.63$, p=0.429; and for EO 61.3 versus 38.7%, $\chi_{1}^{2}=1.58$, p=0.209; Figure 3), we did not find any statistically significant differences. There was also no significant predominance of larval preference for plants with young damaged leaves (for seedlings; and for EO, Figure 3) or mature damaged leaves (for seedlings $\chi_{1}^{2}=2.03$, p=0.155; and for EO $\chi_{1}^{2}=3.45$, p=0.06; Figure 2) compared to undamaged plants. Although not significant, the percentage of larvae choosing plants with young damaged leaves (for seedlings 65.0% and EO 66.7%) was higher than that of the undamaged plants (for seedlings 35.0% and for EO 33.3%). There was also a predominance of choice of larvae for plants with young damaged leaves (for seedlings 80% and EO 73.3%) in comparison to plants with mature damaged leaves (for seedlings 20% and EO 26.7%), showing a significant difference ($\chi_{1}^{2}=13.23$, p<0.001; and for EO $\chi_{1}^{2}=6.53$, p=0.010; Figure 3).

**Figure 4:**
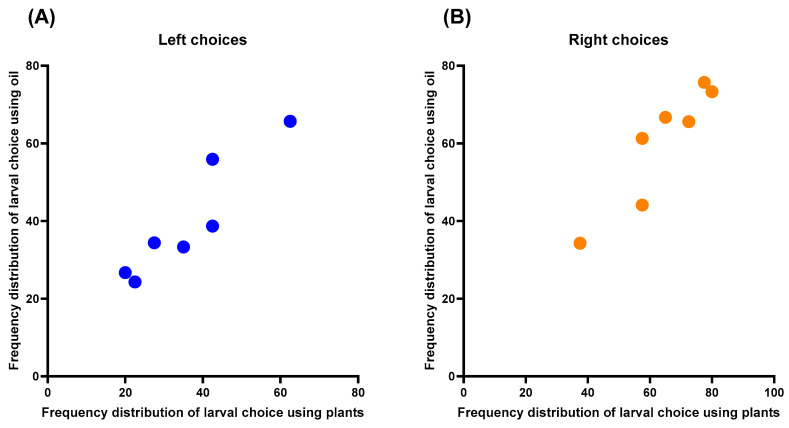
Correlation between the frequency distributions of *Chrysoperla externa* larval choices to *Eucalyptus urograndis* using seedlings and essential oils for the (**A**) left and (**B**) right arms.

The results of the study revealed a remarkable similarity between the values obtained for the left and right sides of the plants and EOs. This finding was supported by Spearman Correlation analysis, which demonstrated a high correlation between the left (seedlings versus EOs; r_s_ = 0.92, p = 0.0052; Figure 4A) and right (seedlings versus EOs; r_s_ = 0.92, p = 0.0052; Figure 4B) choices, suggesting that the volatile compounds emitted by the plant are related to those emitted by the EOs.

## 4. Discussion

### 4.1. EO Extraction

Approximately 17,500 aromatic species of higher plants, mainly from the families Myrtaceae, Lauraceae, Lamiaceae, and Asteraceae, produce essential oils [65]. Among the species of the Myrtaceae family, the genus *Eucalyptus* has more than 200 species with essential oil production in its leaves [66,67], but fewer than 20 species are exploited industrially [68]. *Eucalyptus urograndis* is not included in the main list of essential oil-producing species of *Eucalyptus*, probably because of its low extraction yield, which does not justify the commercial exploitation of their essential oils. Pereira et al. [69] collected leaves at random points in the crown of adult *E. urograndis* and obtained a yield of 1.56%. The result found by this author was expressive when compared to the three species with the highest yield in Brazil, *E. citriodora* Hook, *E. globulus* Labill, and *E. staigeriana* F.Muell, with 1–1.6, 1.7–2, and 1.4%, respectively [67]. However, other studies have yielded results like those obtained in the present study for mature leaves. Goldbeck et al. [70] used leaves from the canopy of *E. urograndis* individuals 19 months old and found a 0.29% yield for essential oil extraction. Bonora [71] evaluated mature *E. urograndis* leaves and obtained a yield of 0.20%.

In our study, the yield of essential oils from mature leaves of *E. urograndis* was not high compared to that from other eucalyptus species. In contrast, the essential oil yield of young leaves was considered moderate according to other studies. Silva et al. [72] evaluated the yield of 11 *Eucalyptus* species, with *E. citriodora*, *E. viminalis* Labill, and *E. globulus* achieving the best results, 1.70, 1.56, and 1.07%; the worst results were found for *E. pellita* F. Muell and *E. cloeziana* F. Muell, being 0.00% and 0.12%, respectively. In an evaluation of essential oils from 12 *Eucalyptus* species, Bonora [71] found higher yields in *E. staigeriana* F. Muell and *E. citriodora* (1.6 and 1.3%, respectively) and lower yields in *Corymbia ptychocarpa* F. Muell and *E. saligna* Smith (0.021 and 0.11%, respectively).

There is still no clear trend regarding the influence of the age of *E. urograndis* leaves on the production of essential oils, and further studies are needed to clarify this topic [72]. In this sense, the results of this study have a significant contribution, since variation in yield and a greater production of essential oil were found in young leaves, corroborating studies by Silvestre et al. [73] and Li et al. [74], who found the higher yield of essential oil in young leaves of *E. globulus* and *E. nitens* (H.Deane & Maiden) Maiden, respectively.

### 4.2. EO Chemical Composition

The GCMS studies led to the chemical identification of 32 compounds, which is higher than that reported in other studies, such as Goldbeck et al. [70], Bonora [71], Araújo et al. [75], and [69], who identified 21, 17, 10, and 10 compounds, respectively. This variation in identification between the studies is probably linked to differences in the extraction methodology as well as in the plants used in the analysis. According to Darrow and Bowers [76], despite the existence of genetic control, environmental and intrinsic factors such as seasonality, competition, and physiology of plants may influence the total content and proportions of plant secondary metabolite compounds.

Araújo et al. [75] and Goldbeck et al. [70] found a predominance of oxygenated monoterpenes and oxygenated sesquiterpenes in their essential oil studies *E. urograndis* leaves, corroborating the results found in this study. Vitti and Brito [67] also reported the predominance of terpenic compounds, monoterpenes, and sesquiterpenes in essential oils. According to Harbone [77], these compounds are related to plant metabolic functions, which can be found in hormones and membrane structures, and according to Andrew et al. [78], Lawler et al. [79], and Marsh et al. [80] are important for direct and indirect interactions between herbivores and other organisms.

The predominance of eucalyptol, α-terpineol, and α-terpinyl acetate found in this study agrees with the results of Bonora [71]. This study was carried out with mature leaves of *E. urograndis*, and the compounds previously mentioned had a higher concentration (eucalyptol, α-terpineol, and α-terpinyl acetate with 17.7, 17.8, and 15.6%, respectively). This variation between compounds was also corroborated by Dellacassa and Moyna [81], who clarified that the existence of qualitative variations among individuals of the same species is common due to genetic, environmental, and leaf type selection, as well as differences in extraction techniques and equipment used in the analysis of essential oils.

The olfactometry tests demonstrated that the leaves of *E. urograndis* responded to simulated herbivory by changing the concentration of the compounds in its essential oil, corroborating the findings of other studies that found changes in leaf chemical composition and predators’ choice [82,83]. In addition, such responses influenced the behavior of *C. externa*, as demonstrated in other studies, which stated that behavioral observations and chemical analyses strongly suggest that the induced volatiles of plants play a fundamental role in the location of the host or prey by natural enemies [83,84,85]. In addition, further research must also investigate the effects of plant volatiles on herbivores, since some studies showed that this guild may change their behavior choice in the presence of some compounds [86,87].

### 4.3. Simulated Herbivory and Olfactometry Tests

Our results demonstrate that *C. externa* larvae show a preference for situations in which volatile organic compounds of *E. urograndis* leaves are present in the seedlings and EOs, indicating that these compounds may play a signaling role in this insect. Other *Chrysoperla* species have shown the same behavior in other studies. For instance, Salamanca et al. [50] demonstrated the attraction of adults of *C. externa* to the volatiles of *Coriandrum sativum* L. (Apiaceae) when isolated and in the presence of flowers of *Rosa hybrida* L. (Rosaceae). In addition, Resende et al. [51] found that adults of *C. externa* had different choices for volatile coriander (*C. sativum* L.) and fennel (*Foeniculum vulgare* Mill.). In the present study, unmated adults were attracted to coriander, whereas mated adults were attracted to fennel. Linalool is one of the main components of coriander essential oils [88] and is also found in the essential oils of damaged young leaves of *E. urograndis*. Some studies have reported that herbivore attacks in other cultures, such as aphids in broad beans [89] and *Pandemis heparana* moths in apples [90], stimulate the release of linalool. The damage to the leaves triggered different responses in the larvae of *C. externa*, with a preference for the odors emitted by young leaves. In this sense, our damaged young leaf group presented a higher linalool content than the other groups, which may indicate that linalool could be a potential signaling compound for *Chrysoperla* species during their foraging time, as our unmated adults presented the same preference.

In our study, only predator insects and plants were used to identify the role of volatiles in plant–predator interactions. Our results showed that the larvae of *C. externa* made choices according to the different types of volatiles of *E. urograndis*, without the participation of prey and other elements. Other studies have shown that chrysopid olfactory preferences are strongly linked to volatile emission profiles in tests, and that species in this group can identify and separate volatiles emitted by plants from those emitted by prey. For instance, Zhu et al. [91], *Chrysoperla carnea*, and *Chrysopa oculata* Say (Neuroptera: Chrysopidae) presented different choices when in contact with the volatiles of alfalfa (*Medicago sativa* L.) and pheromones from the aphid *Acyrthosiphon pisum* Harris (Hemiptera: Aphididae). In their study, *C. carnea* was attracted to the volatiles of alfalfa and *C. oculata* by aphid volatiles. In the same sense, Reddy [92] identified the preference of adults of *C. carnea* to the volatile of eggplant (*Solanum melongena* L., Solanaceae), okra (*Abelmoschus esculents* L., Malvaceae), and pepper (*Capsicum annum* L., Solanaceae) leaves subjected to simulated herbivory. This is probably linked to the fact that predator insects do not depend on host plants only to find suitable prey, and the decision to interact with host plants is not always dependent on the prey’s presence [93]. Previous works have shown that predators respond to the chemical clues emitted by plants both for locating their prey and for the consumption of elements produced by plants to supplement their diet, like “honeydew” and extrafloral nectar [94,95,96].

The olfactometry tests indicated a variation between the volatiles emitted by young and mature leaves, which reinforces the influence of the plant emission profile on insect behavior. Some plants, such as *E. urograndis*, present an inverse relationship between age and the production of indirect defenses, resulting in changes in the interactions between plants, herbivores, and their natural enemies throughout the development of plant structures [97]. The ontogenetic stage of leaves has been shown to be an important determinant of leaf EO production [98,99,100,101], and is usually linked to the preference of insects for young leaves due to aspects related to oviposition and nutrition [102,103].

## 5. Conclusions

Although our methods do not perfectly emulate real field herbivory conditions, our results strongly indicate a preference for *C. externa* over the odor of young leaves with damage to *E. urograndis*. Considering only the variations in eucalyptol, α-terpineol, and α-terpinyl acetate after the damage in young and mature leaves, there is an inversion in the concentration between eucalyptol (which decreased) and α-terpinyl acetate (which increased) with the damage caused to the leaf, whether young or mature. In addition, the essential oil content of young leaves was higher than that of the mature leaves. From this, an important observation can be highlighted: the content of volatile compounds may have influenced the attraction of *C. externa*, because when there are options between clean air and volatiles, *C. externa* larvae prefer volatiles. The higher the volatile content, the greater the attraction. Therefore, in the test of the choice between young and mature leaves with damage, *C. externa* larvae preferred young leaves with damage. Another important observation must be highlighted: the inversion observed in the content of eucalyptol (which decreased) and α-terpinyl acetate (which increased) may be due to the damage caused to the leaves, since this was observed in young and mature leaves. This damage may have influenced the biosynthesis of α-terpinyl acetate, a more volatile compound than eucalyptol. From the observation of larval choice between young leaves with damage and young and mature leaves without damage, the preference of the larvae was for the volatiles emitted by the young leaves with damage. This indicates that some differences may be present in the volatiles that act as major attractors for *C. externa* larvae. The inversion in the content between eucalyptol (which decreased) and α-terpinyl acetate (which increased) may explain this behavior of *C. externa*.

In summary, the practical applications of this study lie in biological pest control through the use of biopesticides and organic chemicals. This emphasizes the use of natural products to attract predators as an alternative to conventional synthetic pesticides used in pest control, an approach that promotes sustainability, for example, in organic farming, compared to conventional practices [104].

## Figures and Tables

**Table 1:** Yield of the essential oil (EO) extraction process by hydrodistillation of *Eucalyptus urograndis*.

Samples	Mass of Leaves (g)	Moisture Content (%)	Mass of EO (mg)	Yield (%)
Young leaves without damage	50.0 ± 0.2	60.7 ± 2.42	99.5 ± 0.3	0.50 ± 0.02
Young leaves with damage	50.0 ± 0.2	60.7 ± 2.42	100.4 ± 0.6	0.50 ± 0.03
Mature leaves without damage	50.0 ± 0.2	55.37 ± 3.16	82.0 ± 0.1	0.37 ± 0.05
Mature leaves with damage	50.0 ± 0.2	55.37 ± 3.16	65.4 ± 0.7	0.29 ± 0.03

**Table 2:** Chemical composition (TIC, %) of essential oils from *Eucalyptus urograndis* leaf groups.

Peak	Compound	TIC (%)			
		Young Leaves without Damage	Young Leaves with Damage	Mature Leaves without Damage	Mature Leaves with Damage
01	Eucalyptol	28.16	10.36	20.33	7.59
02	Linalool oxide <*cis*-> (furanoid)	0.25	0.20	0.19	0.13
03	Linalool oxide <*trans*-> (furanoid)	0.29	0.19	0.22	0.16
04	Linalool	0.60	0.57	0.57	0.49
05	NI	0.40	0.06	0.1	0.05
06	Fenchol<endo->	0.63	0.54	0.69	0.56
07	Campholenal<alpha->	0.26	0.06	0.18	0.16
08	Borneol	1.31	1.80	2.08	2.38
09	Terpinen-4-ol	0.99	1.18	1.43	1.38
10	ρ-Cymen-8-ol	1.17	0.85	0.55	0.50
11	α-Terpineol	10.85	11.90	11.49	13.73
12	NI	1.48	0.55	0.58	0.48
13	2-Hydroxy-1,8-cineole	3.08	1.68	1.59	1.46
14	Neral Carvone *	0.48	0.27	0.27	0.23
15	Geraniol	0.55	0.76	0.90	1.04
16	Geranial	1.14	0.55	0.52	0.58
17	NI	1.48	1.02	0.94	0.92
18	NI	1.52	0.74	1.12	1.13
19	Verbenyl acetate <*trans*->	0.18	0.21	0.17	0.19
20	Exo-2-hydroxycineole acetate	0.53	0.61	0.48	0.56
21	α-Terpinyl acetate	14.24	21.06	17.62	21.96
22	NI	0.35	0.33	0.34	0.37
23	Alpha-copaene	0.27	0.40	0.30	0.38
24	Geranyl acetate	1.67	2.26	1.65	1.89
25	NI	0.82	0.33	0.32	0.48
26	Carvone hydrate	1.27	1.53	1.49	1.55
27	NI	1.02	1.15	1.20	0.91
28	Aromadendrene	0.70	1.05	0.49	0.71
29	NI	1.10	1.11	1.08	1.38
30	NI	1.70	2.02	2.40	2.61
31	NI	0.59	0.73	0.59	0.65
32	Geranyl isobutyrate	2.26	3.60	3.22	4.44
33	NI	1.13	1.59	1.25	1.27
34	Flavesone	0.39	0.71	0.49	0.67
35	8-Acetoxy-carvotanacetone	2.84	3.99	3.49	3.61
36	Spathulenol ** Caryophyllene oxide ** Globulol **	7.25	12.20	10.75	12.37
37	Viridiflorol	0.90	1.55	2.14	2.82
38	NI	0.49	0.83	0.77	0.94
39	Humulene epoxide II	0.28	0.48	0.35	0.55
40	Isoleptospermone	1.58	2.64	2.39	3.21
41	NI	2.52	4.64	1.88	2.43
42	5-Hydroxy-isobornyl-isobutanoate	1.16	1.62	1.29	0.97
	Total identified (%)	85.28	84.82	87.33	86.27

TIC: Total ion chromatogram. NI: not identified. * Carvone mixed with Neral. ** Caryophyllene oxide and Globulol mixed with Spathulenol.

**Table 3:** Variation in the percentage of total ion chromatograms of some compounds in the essential oils of *Eucalyptus urograndis* according to leaf ontogeny and simulated herbivory treatment.

Peak	Compounds	TIC (%) Average					
		Young Leaves without Damage	Young Leaves with Damage	Difference (%)	Mature Leaves without Damage	Mature Leaves with Damage	Difference (%)
01	Eucalyptol	28.16	10.36	−63.2	20.33	7.59	−62.7
11	α-Terpineol	10.85	11.90	9.6	11.49	13.73	19.5
21	α-Terpinyl acetate	14.24	21.06	47.9	17.60	22.00	25.0
28	Aromadendrene	0.70	1.05	50.0	0.49	0.71	44.9
42	5-Hydroxy-isobornyl isobutanoate	1.16	1.62	39.7	1.29	0.97	−24.8

TIC: Total ion chromatogram.

## Data Availability

The data are publicly available in the Supplementary Materials.

## Supplementary Materials

The following supporting information can be downloaded at: https://www.mdpi.com/article/10.3390/plants13162192/s1, Figure S1: Chromatographic profile of essential oils from *Eucalyptus urograndis* leaves (5.0 to 40.0 min) showing a higher concentration of compounds Eucalyptol (1), α-Terpineol (11), and α-Terpinyl acetate (21); Table S1: Arithmetic index of the identified compounds of the essential oils from young leaves without damage (YL), young leaves with damage (YL/D), mature leaves without damage (ML), and mature leaves with damage (ML/D) of *Eucalyptus urograndis*.
