# Supplementary material for: Green Lacewing Chrysoperla externa Is Attracted to Volatile Organic Compounds and Essential Oils Extracted from Eucalyptus urograndis Leaves
Source: Plants (Basel). 2024 Aug 8;13(16):2192. doi: 10.3390/plants13162192 (PMC11360061; doi:10.3390/plants13162192)
Supplement: Supplementary file 1 [file plants-13-02192-s001.zip › plants-3091788-supplementary.pdf]

SUPPLEMENTARY MATERIAL

Figure S1. Chromatographic profile of essential oils from *Eucalyptus urograndis* leaves (5.0 to 40.0 minutes) showing a higher concentration of compounds Eucalyptol (1), α-Terpineol (11), and α-Terpinyl acetate (21).

a) young leaves without damage

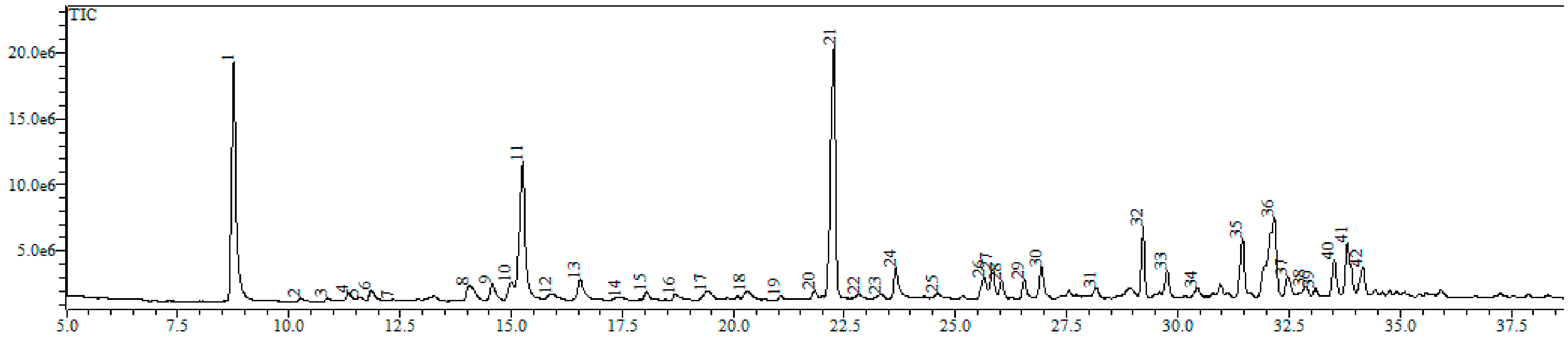

b) young leaves with damage (YL/D)

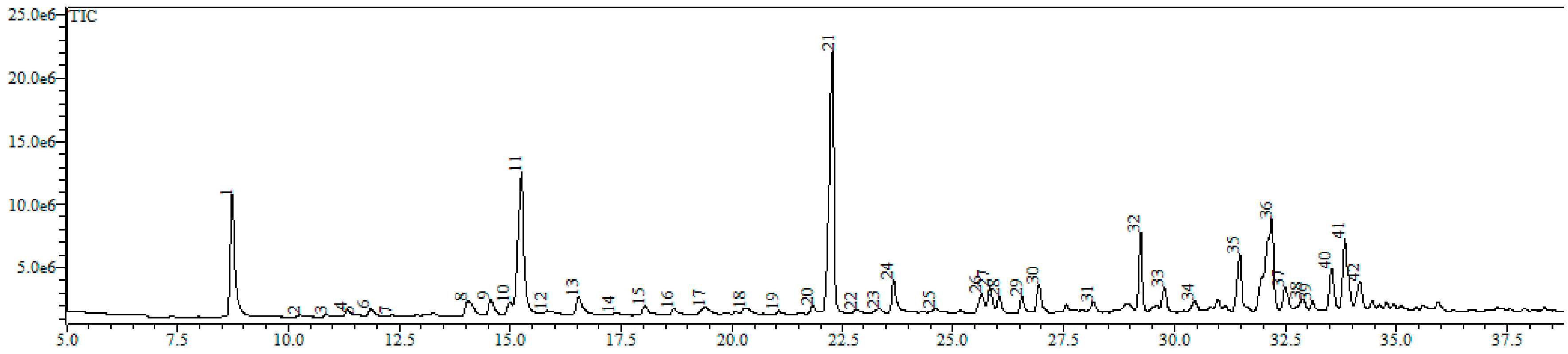

c) mature leaves without damage

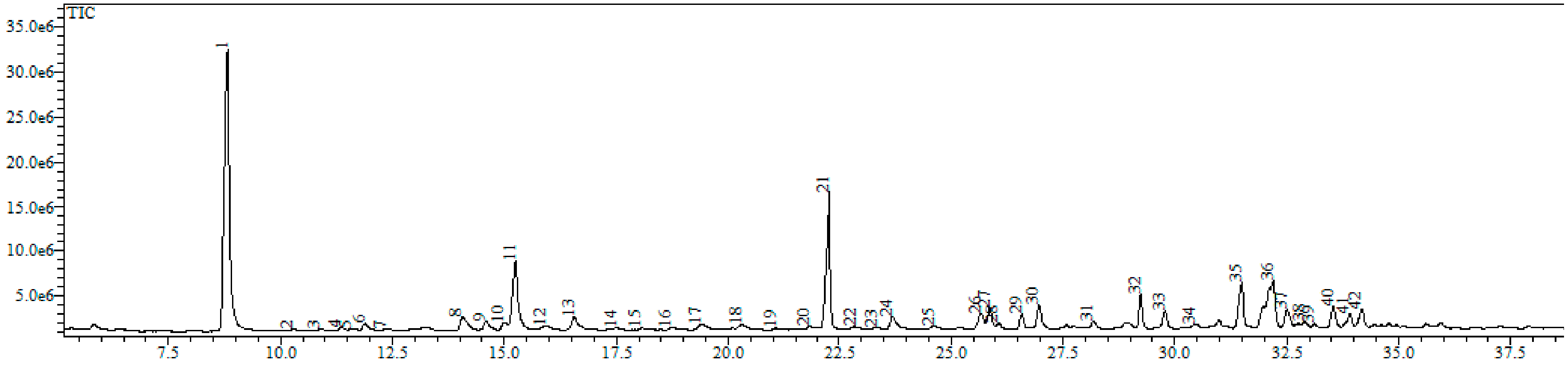

d) mature leaves with damage

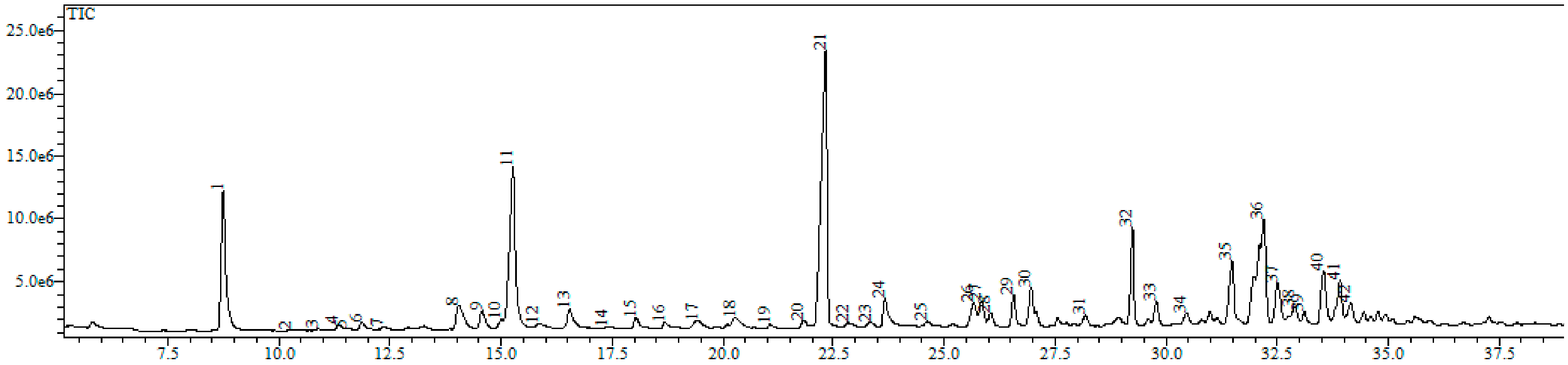

Table S1. Arithmetic index of the identified compounds of the essential oils from young leaves without damage (YL), young leaves with damage (YL/D), mature leaves without damage (ML), and mature leaves with damage (ML/D) of *Eucalyptus urograndis*.

Peak	Compound	Sample	Retention time (min)	AI[a] Literature	AI Calculated	SI[b]	Library
1	Eucalyptol	YL	8.8	1026	1026	97	Nist08
		YL/D	8.7	1026	1024	97	Wiley229
		ML	8.8	1026	1026	97	Nist08
		ML/D	8.7	1026	1024	97	Wiley229
2	Linalool oxide <*cis*-> (furanoid)	YL	10.3	1067	1061	94	Shim2205
		YL/D	10.3	1067	1062	91	Wiley229
		ML	10.3	1067	1062	90	Nist08
		ML/D	10.3	1067	1062	92	Wiley229
3	Linalool oxide <*trans*-> (furanoid)	YL	10.9	1084	1076	91	Wiley229
		YL/D	10.9	1084	1076	90	Wiley229
		ML	10.9	1084	1076	93	Wiley229
		ML/D	10.9	1084	1076	93	Wiley229
4	Linalool	YL	11.3	1095	1085	90	Shim2205
		YL/D	11.3	1095	1086	90	Shim225

		ML	11.4	1095	1088	94	Wiley139
		ML/D	11.3	1095	1086	94	Wiley229
5	Not identified	YL	11.6	-	-	-	-
		YL/D	11.5	-	-	-	-
		ML	11.6	-	-	-	-
		ML/D	11.5	-	-	-	-
6	Fenchol <endo->	YL	11.9	1114	1100	90	Shim2205
		YL/D	11.9	1114	1100	92	Shim225
		ML	11.9	1114	1100	94	Shim2205
		ML/D	11.9	1114	1100	95	Shim2205
7	Campholenal <α->	YL	12.4	1122	1114	94	Shim2205
		YL/D	12.3	1122	1111	80	Shim225
		ML	12.4	1122	1114	94	Shim2205
		ML/D	12.3	1122	1111	94	Shim2205
8	Borneol	YL	14.0	1165	1157	97	Wiley229
		YL/D	14.0	1165	1157	92	Shim225
		ML	14.1	1165	1160	97	Wiley229
		ML/D	14.1	1165	1160	95	Wiley229
9	Terpinen-4-ol	YL	14.6	1174	1173	92	Wiley229
		YL/D	14.6	1174	1173	90	Shim225
		ML	14.6	1174	1173	78	Nist08

		ML/D	14.6	1174	1173	85	Nist08
10	p-Cymen-8-ol	YL	15.0	1179	1184	90	Wiley229
		YL/D	15.0	1179	1184	90	Wiley229
		ML	15.0	1179	1184	90	Nist08
		ML/D	15.0	1179	1184	88	Nist08
11	α-Terpineol	YL	15.2	1186	1190	97	Wiley229
		YL/D	15.3	1186	1192	95	Wiley229
		ML	15.3	1186	1192	95	Wiley229
		ML/D	15.3	1186	1192	95	Wiley229
12	Verbenone	YL	15.9	1204	1206	92	Wiley229
		YL/D	15.8	-	-	-	-
		ML	15.9	1204	1206	91	Nist08
		ML/D	15.9	-	-	-	-
13	2-Hydroxy-1,8-cineole	YL	16.5	1227	1217	93	Nist08
		YL/D	16.5	1227	1217	94	Nist08
		ML	16.6	1227	1219	93	Nist08
		ML/D	16.5	1227	1217	93	Nist08
14	Neral	YL	17.5	1235	1235	90	Wiley229

		YL/D	17.4	1235	1233	91	Wiley229
		ML	17.4	1235	1233	85	Nist08
		ML/D	17.4	1235	1233	90	Nist08
	Carvone	YL	17.5	1239	1235	91	Wiley229
		YL/D	17.4	1239	1233	80	Wiley229
		ML	17.4	1239	1233	85	Nist08
		ML/D	17.4	1239	1233	90	Nist08
15	Geraniol	YL	18.0	1249	1245	80	Wiley139
		YL/D	18.0	1249	1245	84	Wiley229
		ML	18.1	1249	1246	80	Nist08
		ML/D	18.0	1249	1245	88	Nist08
16	Geranial	YL	18.7	1264	1257	96	Wiley229
		YL/D	18.7	1264	1257	96	Wiley229
		ML	18.7	1264	1257	97	Wiley229
		ML/D	18.7	1264	1257	97	Wiley229
17	Not identified	YL	19.4	-	-	-	-
		YL/D	19.4	-	-	-	-
		ML	19.4	-	-	-	-
		ML/D	19.4	-	-	-	-
18	Not identified	YL	20.3	-	-	-	-
		YL/D	20.3	-	-	-	-
		ML	20.3	-	-	-	-
		ML/D	20.3	-	-	-	-
19	Verbenyl acetate-trans	YL	21.1	1291	1301	79	Shim2205

		YL/D	21.1	1291	1301	74	Nist08
		ML	21.1	1291	1301	80	Shim2205
		ML/D	21.0	1291	1300	80	Shim2205
20	exo-2-Hydroxy-cineole acetate	YL	21.8	1342	1323	87	Nist08
		YL/D	21.8	1342	1323	91	Nist08
		ML	21.8	1342	1323	90	Nist08
		ML/D	21.8	1342	1323	91	Nist08
21	α-Terpinyl acetate	YL	22.3	1346	1338	93	Nist08
		YL/D	22.3	1346	1337	92	Wiley229
		ML	22.3	1346	1338	94	Shim2205
		ML/D	22.3	1346	1338	93	Wiley229
22	Not identified	YL	22.8	-	-	-	-
		YL/D	22.8	-	-	-	-
		ML	22.9	-	-	-	-
		ML/D	22.8	-	-	-	-
23	alpha.-Copaene	YL	23.3	1374	1367	90	Willey229
		YL/D	23.3	1374	1367	87	Willey229

		ML	23.4	1374	1370	86	Willey229
		ML/D	23.3	1374	1367	80	Willey229
24	Geranyl acetate	YL	23.7	1379	1367	90	Wiley229
		YL/D	23.7	1359	1379	93	Wiley229
		ML	23.7	1379	1379	90	Wiley229
		ML/D	23.7	1379	1379	90	Wiley139
25	Not identified	YL	24.6	-	-	-	-
		YL/D	24.6	-	-	-	-
		ML	24.6	-	-	-	-
		ML/D	24.6	-	-	-	-
26	Carvone hydrate	YL	25.6	1422	1429	94	Shim2205
		YL/D	25.7	1422	1432	93	Shim225
		ML	25.7	1422	1432	93	Shim2205
		ML/D	25.7	1422	1432	93	Shim2205
27	Not identified	YL	25.8	-	-	-	-
		YL/D	25.8	-	-	-	-
		ML	25.9	-	-	-	-

		ML/D	25.9	-	-	-	-
28	Aromadendrene	YL	26.0	1439	1440	95	Wiley229
		YL/D	26.1	1439	1442	95	Wiley229
		ML	26.1	1439	1442	94	Wiley229
		ML/D	26.0	1439	1440	93	Wiley229
29	Not identified	YL	26.6	-	-	-	-
		YL/D	26.6	-	-	-	-
		ML	26.6	-	-	-	-
		ML/D	26.6	-	-	-	-
30	Not identified	YL	26.9	-	-	-	-
		YL/D	27.0	-	-	-	-
		ML	27.0	-	-	-	-
		ML/D	27.0	-	-	-	-
31	Not identified	YL	28.2	-	-	-	-
		YL/D	28.2	-	-	-	-
		ML	28.2	-	-	-	-
		ML/D	28.2	-	-	-	-

32	Geranyl isobutyrate	YL	29.2	1514	1518	95	Willey 139
		YL/D	29.2	1514	1518	93	Willey 139
		ML	29.3	1514	1521	93	Shim2205
		ML/D	29.2	1518	1518	94	Willey229
33	Not identified	YL	29.8	-	-	-	-
		YL/D	29.8	-	-	-	-
		ML	29.8	-	-	-	-
		ML/D	29.8	-	-	-	-
34	Flavesone	YL	30.5	1545	1548	82	Shim2205
		YL/D	30.5	1545	1548	81	Shim225
		ML	30.5	1545	1578	81	Shim2205
		ML/D	30.5	1545	1548	84	Shim2205
35	8-Acetoxy-Carvotanacetone	YL	31.4	1564	1569	86	Nist08
		YL/D	31.5	1564	1571	86	Nist08
		ML	31.5	1564	1571	86	Shim2205
		ML/D	31.5	1564	1571	86	Shim2205
36	Spathulenol	YL	32.2	1577	1587	90	Wiley229

		YL/D	32.2	1577	1587	85	Wiley229
		ML	32.2	1577	1587	91	Nist08
		ML/D	32.2	1577	1587	86	Wiley229
	Caryophyllene oxide	YL	32.2	1582	-	87	Wiley229
		YL/D	32.2	1582	-	74	Wiley229
		ML	32.2	1582	-	82	Wiley229
		ML/D	32.2	1582	-	80	Wiley229
	Globulol	YL	32.2	1590	-	75	Wiley229
		YL/D	32.2	1590	-	83	Wiley229
		ML	32.2	1590	-	81	Wiley229
		ML/D	32.2	1590	-	86	Wiley139
37	Viridiflorol	YL	32.5	1592	1594	91	Willey229
		YL/D	32.5	1592	1594	90	Shim2205
		ML	32.5	1592	1594	92	Willey229
		ML/D	32.5	1592	1594	91	Willey229
38	Not identified	YL	32.9	-	-	-	-
		YL/D	32.9	-	-	-	-

		ML	32.9	-	-	-	-
		ML/D	32.9	-	-	-	-
39	Humulene epoxide II	YL	33.1	1608	1609	86	Shim2205
		YL/D	33.1	1608	1609	85	Shim2205
		ML	33.1	1608	1609	83	Shim2205
		ML/D	33.1	1608	1609	85	Nist08
40	Leptospermone<iso->	YL	33.5	1621	1619	85	Shim2205
		YL/D	33.5	1621	1619	90	Shim2205
		ML	33.6	1621	1622	89	Shim2205
		ML/D	33.5	1621	1619	82	Shim2205
41	Not identified	YL	33.8	-	-	-	-
		YL/D	33.8	-	-	-	-
		ML	33.9	-	-	-	-
		ML/D	33.9	-	-	-	-
42	Isobornyl isobutanoate<5-hydroxy->	YL	34.1	1658	1635	83	Shim2205
		YL/D	34.2	1656	1637	83	Shim2205
		ML	34.1	1658	1635	83	Shim2205

		ML/D	34.2	1656	1637	84	Shim2205

Note: [a]Arithmetic index, [b]Identification method by similarity index with mass spectral database: Nist08, Wiley139, Wiley229, Shim225.
